# Changes in primary care physician’s management of low back pain in a model of interprofessional collaborative care: an uncontrolled before-after study

**DOI:** 10.1186/2045-709X-21-6

**Published:** 2013-02-01

**Authors:** Silvano Mior, Brian Gamble, Jan Barnsley, Pierre Côté, Elie Côté

**Affiliations:** 1 Department of Research, Canadian Memorial Chiropractic College, 6100 Leslie Street, Toronto, ON, M2H 3J1, Canada; 2 Private practice, Chatham, ON, Canada; 3 Department of Health Policy, Management & Evaluation, University of Toronto, 155 College Street, 4th Floor, Toronto, Canada; 4 Faculty of Health Sciences, University of Ontario Institute of Technology, 2000 Simcoe Street North, Oshawa, ON, L1H 7K4, Canada

**Keywords:** Interprofessional collaboration, Medication use, Provider behaviour, Chiropractic

## Abstract

**Background:**

Tracking how clinicians treat patients provides an opportunity to explore how the clinical management of common musculoskeletal disorders evolves over time. We present an uncontrolled before-after study of a primary care physician’s management of low back pain and describe how his involvement in an interprofessional collaborative practice was associated with a change in the management of patients with low back pain.

**Method:**

Data from the electronic medical record of one primary care physician who participated in a study of a model of chiropractic-medical collaboration were retrospectively collected. Records of a sample of consecutive patients prior to the start (i.e. pre-study, n = 51) and at the end of the collaborative study (i.e. study, n = 49) were collected.

**Results:**

Demographics were similar in both groups but median number of physician visits (2.5 and 1.0), average prescriptions per patients (1.24 and 0.47), and total number of narcotic prescriptions (14 and 6) differed between pre-study and study groups, respectively. Separate analysis of only the records of low back pain study patients revealed that 61% were referred for chiropractic care during the study period. Patients who were not referred had more neurological deficits and leg pain but back pain severity and average number of prescriptions was about the same. Referred patients in the study group had about 25% fewer physician visits and imaging requests.

**Conclusion:**

Based on this study of a single primary care physician, we hypothesize that doctors may change their prescribing behaviours and consultation rate for patients with low back pain when engaged in interprofessional collaborative care. Further research is required to test this observation in the population.

## Introduction

Chronic musculoskeletal conditions are a significant cause of disability and health care utilization [1,2]. Côté et al. reported that 31% of people with low back or neck pain seek care from both physicians and chiropractors [3]. The nature of this care varies greatly across health care specialities. For example, patients with chronic musculoskeletal conditions who consult a physician and chiropractor take more medication than those who only consult a chiropractor [4]. But it is unknown if there was any level of interprofessional collaboration in the management of patients attending both health care providers. However, evidence suggests that communication between chiropractors and physicians is limited, which may foster fragmentation of care and impact its continuity and quality [5,6].

Evidence also suggests that a multidisciplinary approach to chronic conditions improves patient outcomes and patient and provider satisfaction [7-9]. Empirically, such improvement is founded on care being delivered by the appropriate health care provider with the greatest expertise for a given problem [10]. Health care providers working in primary care settings promoting collaborative practice, as in Family Health Teams (i.e. interprofessional collaborative health care teams delivering coordinated quality care by various health care providers [11]), are well positioned to deliver such multidisciplinary care [12]. For instance, Kopansky-Giles et al. noted clinically important improvements in patients with musculoskeletal complaints who had access to chiropractic care in a hospital-based primary care setting [9].

We present a study of a primary care physician’s management of low back pain and describe how his involvement in an interprofessional collaborative practice was associated with a change in his management of patients with low back pain.

## Method

### Design

This uncontrolled before-after study was nested in a two-year observational study designed to evaluate the implementation of an interprofessional collaborative model [13].

The main observational study had as its objective to describe the implementation of a process-based model upon the professional relationship of chiropractors and primary care physicians, the continuity of patient care, the level of satisfaction of providers and patients, and the reimbursement of chiropractic services. The model involved strategies that addressed four key attributes of collaborative practice: communication (structured clinical notes, educational sessions, newsletters); defined musculoskeletal scope of practice; service delivery (no patient pay, provider capitated payments, access to diagnostic testing); and patient-centred care (patient choice, focus on prevention/supportive care) [12,13]. Participants included primary care physicians, their patients and chiropractors.

### Patients

We formed two case series of consecutive patients with low back pain who presented before and after the implementation of the observational study to one of the study primary care physicians (physician) who belonged to a Family Health Team (FHT) in rural Ontario, Canada. De-identified patient data were collected at two different points in time from the electronic medical record (EMR) of patients who presented with a chief complaint of low back pain (LBP). The first case series included patients who consulted the primary care physician prior to the start of (i.e. pre-study group) and the second included patients who were enrolled in the collaborative study (i.e. study group).

### Data collection and outcome measures

Information extracted from the EMR included the patients’ age, sex and patient-reported subjective rating of pain severity (on scale of 0- to 5, where 0 equated to no pain and 5 was worse pain ever). Our outcome measures included number of physician visits related to the presenting complaint, number and category of medications prescribed and clinical symptoms (location, neurological deficits, presence of co-morbidities).

All data were extracted using discrete patient identifiers and stored on an excel spreadsheet. Data were then entered into a statistical program (SPSS 14.0) and descriptively analyzed. Ethics approval was obtained from the Canadian Memorial Chiropractic College and the University of Toronto.

## Results

There were 51 and 49 available LBP patient records extracted in the pre-study and study groups, respectively (see Table 1). The age of the patients and severity of LBP were similar in the two groups; however, there were more females in the pre-study group. The average number of physician visits during both time periods was about the same but the median number of visits was fewer in the study group compared to the pre-study group.

**Table 1:** A descriptive summary of variables extracted from the EMR comparing pre-study and study patients

**EMR selected variable**	**Pre-study**	**Study**
Number of cases	51	49
Number of females (%)	27 (53)	21 (43)
Mean patient age (sd)	59 (17.6)	59 (13.6)
Severity of presenting condition (median)	2 of 5	2 of 5
Number patients prescribed medication (%)	44 (86.3)	21 (43.9)
Total number of medications prescribed	63	24
Average number of prescriptions (sd)	1.24 (0.71)	0.47 (0.62)
Total number of patient visits	132	101
Average number of physician visits (sd)	2.6 (1.3)	2.1 (1.6)
Median number of physician visits (range)	2.5 (5)	1.0 (6)

Table 2 summarizes the number and type of medications prescribed by the physician to patients in each group. There were twice as many patients in the pre-study group who were prescribed medication compared to the study group. Almost 33% of patients in the pre-study group were concurrently prescribed a second, and 4% a third medication, compared to 6% of patients in study group who received only a second prescription. Despite the similarity in recorded pain severity, there were about 2.6 times more medications prescribed in the pre-study group compared to those in the study group. There were more patients in the pre-study group who received prescriptions for either an NSAID or a Cox 2 inhibitor compared to the study group. Of note, for those receiving a second medication, there was a greater percentage in the pre-study group receiving a narcotic prescription. There were about twice the number of narcotic prescriptions provided to patients in the pre-study group as in the study group.

**Table 2:** Summary of the categories and the number of medications prescribed to patients in both groups

**Category**	**Pre-study group (n=51)**			**Study group (n=49)**		
	**Drug 1**	**Drug 2**	**Drug 3**	**Drug 1**	**Drug 2**	**Drug 3**
NSAID n (%)	12 (23.5)	2 (3.9)	0	8 (16.3)	1 (2.0)	0
Narcotic	7 (13.7)	6 (11.8)	1 (2.0)	5 (9.8)	1 (2.0)	0
Cox 2	12 (23.6)	1 (2.0)	0	6 (12.2)	1 (2.0)	0
SSRI	7 (13.7)	2 (3.9)	0	2 (4.1)	0	0
Other	6 (11.8)	6 (11.8)	1 (2.0)	0	0	0
**Total**	**44 (86.3)**	**17 (33.3)**	**2 (3.9)**	**21 (42.9)**	**3 (6.1)**	**0**

Table 3 shows that the physician referred 61% of patients in the study group for chiropractic services compared to none in the pre-study group. The sex, age and severity of pain of the study patients who were referred were similar to those who were not referred for chiropractic services. A greater percentage of the study group patients who were not referred for chiropractic care had more distal leg radiations and neurological deficits than those who were referred. Study patients who were referred by the physician to the chiropractors also had fewer co-morbidities but the nature of the co-morbidities (e.g. diabetes, depression, cardiovascular disease, rheumatoid arthritis) were about the same as in the not-referred study patients. More patients in the study group who were referred to a chiropractor were prescribed medications but the overall average number of prescription medications was about the same as in the non-referred group. The majority of medications prescribed were NSAIDs and Cox-2 inhibitors. Narcotics were prescribed in about 14% of the patients who were referred for chiropractic services and about 43% who were not referred. Patients referred for chiropractic services had almost 24% fewer physician visits. About 25% fewer patients in the study group who were referred for chiropractic care had imaging compared to the non-referred group.

**Table 3:** Summary of variables from the EMR of study patients who attended for care during the study

**EMR selected variable**	**Not referred**	**Referred to chiropractor**
Number of cases	19	30
Number of female cases (%)	9 (47)	12 (40)
Mean patient age (sd)	60 (16.3)	58 (11.7)
Severity of presenting condition (median)	2 of 5^+^	2 of 5^+^
Low back pain +/− buttock pain (%)	5 (26)	24 (80)
Low back pain + leg radiations (%)	9 (47)	5 (17)
Presence of neurological deficits	11 (58)	12 (40)
Presence of co-morbidities (%)	12 (63)	13 (43)
Medication was prescribed (%)	7(37)	14 (47)
Average number of prescriptions (sd)	0.42 (0.61)	0.50 (0.63)
Average number of physician visits (sd)	2.6 (2.0)	1.8 (1.1)
Imaging was requisitioned (%)	14 (74)	15 (50)

(^+^ Severity graded between 0 and 5, where 5 was most severe.).

## Discussion

We observed significant changes in the primary care physician’s clinical management of patients with low back pain following his involvement in an interprofessional model of collaborative care. The physician prescribed fewer medications to study patients compared to pre-study patients, yet the patients had similar pain severity. This difference may have been related to the physician’s participation in the collaborative model of care that facilitated patient access and choice to an alternative treatment modality, as well as to the educational sessions that highlighted evidence based care and LBP guidelines.

A study conducted by Bishop et al. provides some support for the use of a multi-modal guidelines-based plan in treating low back pain [14]. They compared the outcomes of multi-modal guidelines-based care, which included a chiropractor delivering manipulative care, to that of primary care physician-directed usual care. Interestingly, the use of narcotic analgesic medications was 78% in the usual care group, compared to 0% in the multi-modal group. Despite this difference in medication use, improvement in bodily pain was comparable between the two groups. However, they found the guideline-based multi-modal treatment to be associated with significantly greater improvement in condition-specific functioning. This appears to support our results that patients seeing both a chiropractor and a primary care physician in an evidence based collaborative-like setting use less medication.

The study group patients who were referred by the physician for chiropractic care had fewer physician visits than non-referred patients, the latter having the same average number of visits as the pre-study group. However, the non-referred study patients tended to have more radiating pain and neurological deficits, suggesting a more complicated condition that could possibly explain the greater number of physician visits. It is possible that the physician was pre-selecting patients for chiropractic referral based on his experience participating in the study or applying evidence based care.

Patients in the study group, who were referred, had fewer subsequent primary care physician visits suggesting a possible opportunity for primary care physicians to see other patients, thus decreasing their wait lists. The visits to the chiropractor were not tracked and hence the overall impact of the referral on the health care system was not considered.

Previous studies have suggested that socioeconomic factors, such as employment, play a significant role in the use of prescription medications and alternative treatments [15,16]. Recently, opioid use among socioeconomically disadvantaged patients in Ontario has increased substantially [17]. Similarly, patients using opioids were more likely to be unemployed, implying economic disadvantage [18]. As well, higher socioeconomic status was found to be related to decreased use of analgesics and sedatives [16]. The authors suggested that a reason for the difference in medication use was that such patients could afford paying for alternative therapies. Participating in a collaborative model of care with access to no cost alternative therapy appeared to decrease the frequency of prescribing regardless of whether the patient was referred. This decrease could have been attributed to increased primary care physician choice to access alternative care, more discriminating prescription use of medications by the primary care physician, or simply primary care physician involvement in the study, which allowed patient referral without considering ability to pay for chiropractic services.

The decreased use of medications in patients referred for chiropractic services has been reported previously [19-21]. Rhee et al. conducted a retrospective study using administrative claims data from a sample of 13,670 LBP patients [20]. They reported patients seeing a chiropractor were found to be less likely to use narcotic medications, supporting a previous finding that chiropractic care could be used as “a substitute treatment to pain medication and other health care services in patients with LBP” [20: 2610].

Finally, studies have reported that patients with chronic musculoskeletal conditions tend to visit physicians and chiropractors, and take more medications than patients seeing only a chiropractor [3,4]. It may be that such visits to both providers occurred with limited interprofessional collaboration. The findings reported herein suggest that patients with musculoskeletal conditions whose physician participated in a collaborative model of care were prescribed fewer medications, frequently referred to another health care provider, and had fewer physician visits. Also, fewer patients were prescribed two and three medications for low back pain, thus decreasing potential drug-drug interactions. This is important because approximately one third of patients taking opioids for chronic low back pain are at an increased risk of an adverse drug-drug interaction [22]. Furthermore, this enhanced level of interprofessional collaboration led to greater communication between providers and improved continuity of care, i.e. care delivered in a coordinated and timely manner [23]. These findings raise interesting questions about how interprofessional collaborative care can change provider behaviour and influence the overall utilization of health care resources and quality of care.

Evidence suggests improving patient care depends partly on the ability of health care providers to change their behaviour [24]. The application of a particular behaviour change theory may provide understanding of the array of factors that influence such change. The theory of planned-behaviour hypothesizes that an individuals’ perceived control over, and their intention to perform a behaviour are determining factors to its engagement [25]. The strength of this intention is influenced by the attitudes and beliefs towards a particular behaviour, the normative beliefs, and motivation to comply. The physician in our study was a site champion, involved in planned site administrative and education meetings, and had previous interprofessional experience, variable factors that could influence intention and behaviour change. Further work may help inform how behavioural theory can influence interprofessional collaboration and enhance the delivery of quality patient-centred care.

There are significant limitations to an uncontrolled before-after study. In our study, the data were extracted from a consecutive sample of patients from the EMRs of a single primary care physician who may have been biased due to his involvement in the study. As such, the results noted could also have been favourably confounded by the Hawthorne effect [26], where the physician’s behaviour was modified by their participation. Although this is a fundamental concern with our selected study design, the noted observed changes in the number of medication prescriptions and the referral patterns of study patients suggests that the implementation of the collaborative model did have some influence upon the physician’s management of low back pain patients. In addition, coding bias or recording errors of various aspects of the patients’ visit and medication used may have occurred. However, the frequency of medication prescription and trend to decreased drug use are similar to previously reported larger studies.

## Conclusion

This uncontrolled before-after study suggests that interprofessional collaborative practice between primary care physicians and chiropractors, where patients were provided optional full access to chiropractic services, may influence primary care physician’s management of and prescribing pattern for patients with low back pain. Further research is required to confirm such findings. If similar findings are found in a more robust study design, they may have important health policy implications related to collaboration in primary care, primary care physician workloads, and accessible health care.

## Competing interests

The authors declare that they have no competing interests.

## Authors’ contributions

SM and BG conceived the study, conceptualized the data and drafted the manuscript. JB and PC participated in its design and analyzed and interpreted the data. EC reviewed and summarized the literature and helped draft the manuscript. All authors read and approved the final manuscript.

## References

[B1] Hawker G Williams J, Badley EM Epidemiology of arthritis and osteoporosis Patterns of Health Care in Ontario: Arthritis and Related Conditions 1998 Toronto: Institute for Clinical Evaluative Sciences 1 10

[B2] Von Korff M Lin EH Fenton JJ Saunders K Frequency and priority of paint patients' health care use Clin J Pain 2007 23 400 408 10.1097/AJP.0b013e31804ac020 17515738

[B3] Côté P Cassidy JD Carroll L The treatment of neck and back pain: Who seeks care? Who goes where? Med Care 2001 39 956 967 10.1097/00005650-200109000-00006 11502953

[B4] Hurwitz EL Chiang L A comparative analysis of chiropractic and general practitioner patients in North America: findings from the joint Canada/United States survey of health, 2002–03 BMC Health Serv Res 2006 6 49 http://www.biomedcentral.com/1472-6963/6/49 10.1186/1472-6963-6-49 16600038 PMC1458338

[B5] Mainous AG 3rd Gill JM Zoller JS Wolman MG Fragmentation of patient care between chiropractors and family physicians Arc Fam Med 2000 9 446 450 10.1001/archfami.9.5.446 10810950

[B6] Greene BR Smith M Allareddy V Haas M Referral patterns and attitudes of primary care physicians towards chiropractors BMC Complement Altern Med 2006 6 5 http://www.biomedcentral.com/1472-6882/6/5 10.1186/1472-6882-6-5 16509963 PMC1456998

[B7] Garner MJ Birmingham M Aker P Moher D Balon J Keenan D Manga P Developing integrative primary healthcare delivery: adding a chiropractor to the team Explore 2008 4 18 24 10.1016/j.explore.2007.10.003 18194787

[B8] Maddison P Jones J Breslin A Barton C Fleur J Lewis R McSweeney L Norgain C Smith S Thomas C Tillson C Improved access and trageting of musculoskeletal services in northwest Wales: trageted early access to musculoskeletal services (TEAMS) programme BMJ 2008 329 1325 1327 15576743 10.1136/bmj.329.7478.1325 PMC534845

[B9] Kopansky-Giles D Vernon H Boon H Steinman I Kelly M Kachan N Inclusion of a CAM therapy (*chiropractic care*) for the management of musculoskeletal pain in an integrative, inner city, hospital-based primary care setting J Alter Med Res 2010 2 61 74

[B10] Goldman J Meuser J Rogers J Lawrie L Reeves S Interprofessional collaboration in family health teams Can Fam Physician 2010 56 368 74 PMC2954101 20944025

[B11] Skolardis S Oandasan I Kimpton S Family health teams: can health professionals learn to work together? Can Fam Physician 2007 53 1198 1199 17872817 PMC1949304

[B12] Mior SA Barnsley J Boon H Ashbury FD Haig R Designing a framework for the delivery of collaborative musculoskeletal care involving chiropractors and physicians in community-based primary care J Interprof Care 2010 24 678 689 10.3109/13561821003608757 20441400

[B13] Mior S Barnsley J Boon H Cote P Gamble B Haig R Hayes R Patient Outcomes in a Model of interprofessional Patient-Centered Collaborative practice-depth Case Study Design. In Proceedings of WFC’s 9th Biennial Congress and ECU’s 75th Anniversary Convention: 17–19 May 2007; Vilamoura 2007 Toronto: World Federation of Chiropractic 209 212

[B14] Bishop PB Quon JA Fisher CG Dvorak MF The Chiropractic Hospital-based Interventions Research Outcomes (CHIRO) study: a randomized controlled trial on the effectiveness of clinical practice guidelines in the medical and chiropractic management of patients with acute mechanical low back pain Spine J 2010 10 1055 1064 10.1016/j.spinee.2010.08.019 20889389

[B15] Northcott HC Bachynsky JA Concurrent utilization of chiropractic, prescription medicines, non-prescription medicines and alternative health care Soc Sci Med 1993 37 431 435 10.1016/0277-9536(93)90273-7 8356491

[B16] Haetzman M Elliott AM Smith BH Hannaford P Chambers WA Chronic pain and the use of conventional and alternative therapy Fam Pract 2003 20 147 154 10.1093/fampra/20.2.147 12651788

[B17] Gomes T Juurlink DN Dhalla IA Mailis-Gagnon A Paterson JM Mamdani MM Trends in opioid use and dosing among socio-economically disadvantaged patients Open Med 2011 5 13 22 PMC3205807 22046214

[B18] Fitzcharles MA Ste-Marie PA Gamsa A Ware MA Shir Y Opioid use, misuse, and abuse in patients labeled as fibromyalgia Am J Med 2011 124 955 960 10.1016/j.amjmed.2011.05.031 21962316

[B19] Vogt MT Kwoh K Cope DK Osial TA Culyba M Starz TW Analgesic usage for low back pain: impact on health care costs and service use Spine 2005 30 1075 1081 10.1097/01.brs.0000160843.77091.07 15864162

[B20] Rhee Y Taitel MS Walker DR Lau DT Narcotic drug use among patients with lower back pain in employer health plans: a retrospective analysis of risk factors and health care services Clin Ther 2007 29 2603 2612 10.1016/j.clinthera.2007.12.006 18164925 PMC2747728

[B21] Gurden M Morelli M Sharp G Baker K Betts N Bolton J Evaluation of a general practitioner referral service for manual treatment of back and neck pain Prim Health Care Res Dev 2012 30 1 7 10.1017/S1463423611000648 22284899

[B22] Pergolizzi JV Jr Labhsetwar SA Puenpatom RA Joo S Ben-Joseph RH Summers KH Prevalence of exposure to potential CYP450 pharmacokinetic drug-drug interactions among patients with chronic low back pain taking opioids Pain Pract 2011 11 230 239 10.1111/j.1533-2500.2010.00413.x 20807350

[B23] Haggerty JL Reid RJ Freeman GK Starfield BH Adair CE McKendry R Continuity of care: multidisciplinary review BMJ 2003 327 1219 1221 10.1136/bmj.327.7425.1219 14630762 PMC274066

[B24] Michie S Designing and implementing behaviour change interventions to improve population health J Health Serv Res Policy 2008 13 Suppl 3 64 69 18806194 10.1258/jhsrp.2008.008014

[B25] Walker AE Grimshaw JM Armstrong EM Salient beliefs and intentions to prescribe antibiotics for patients with a sore throat Br J Health Psychol 2001 6 347 360 10.1348/135910701169250 12614509

[B26] Grimshaw J Campbell M Eccles M Steen N Experimental and quasi-experimental designs for evaluating guideline implementation strategies Fam Pract 2000 17 Suppl 1 S11 16 10735262 10.1093/fampra/17.suppl_1.s11

